# Human Herpesvirus 8 Infects and Replicates in Langerhans Cells and Interstitial Dermal Dendritic Cells and Impairs Their Function

**DOI:** 10.1128/JVI.00909-17

**Published:** 2017-09-27

**Authors:** Giovanna Rappocciolo, Mariel Jais, Paolo A. Piazza, Diana C. DeLucia, Frank J. Jenkins, Charles R. Rinaldo

**Affiliations:** a Graduate School of Public Health, University of Pittsburgh, Pittsburgh, Pennsylvania, USA; b School of Medicine, University of Pittsburgh, Pittsburgh, Pennsylvania, USA; University of Southern California

**Keywords:** DC-SIGN, dermal dendritic cells, ephrin A2 receptor, HHV-8, Kaposi's sarcoma-associated herpesvirus, Langerhans cells, langerin

## Abstract

The predominant types of dendritic cells (DC) in the skin and mucosa are Langerhans cells (LC) and interstitial dermal DC (iDDC). LC and iDDC process cutaneous antigens and migrate out of the skin and mucosa to the draining lymph nodes to present antigens to T and B cells. Because of the strategic location of LC and iDDC and the ability of these cells to capture and process pathogens, we hypothesized that they could be infected with human herpesvirus 8 (HHV-8) (Kaposi's sarcoma [KS]-associated herpesvirus) and have an important role in the development of KS. We have previously shown that HHV-8 enters monocyte-derived dendritic cells (MDDC) through DC-SIGN, resulting in nonproductive infection. Here we show that LC and iDDC generated from pluripotent cord blood CD34^+^ cell precursors support productive infection with HHV-8. Anti-DC-SIGN monoclonal antibody (MAb) inhibited HHV-8 infection of iDDC, as shown by low expression levels of viral proteins and DNA. In contrast, blocking of both langerin and the receptor protein tyrosine kinase ephrin A2 was required to inhibit HHV-8 infection of LC. Infection with HHV-8 did not alter the cell surface expression of langerin on LC but downregulated the expression of DC-SIGN on iDDC, as we previously reported for MDDC. HHV-8-infected LC and iDDC had a reduced ability to stimulate allogeneic CD4^+^ T cells in the mixed-lymphocyte reaction. These results indicate that HHV-8 can target both LC and iDDC for productive infection via different receptors and alter their function, supporting their potential role in HHV-8 pathogenesis and KS.

**IMPORTANCE** Here we show that HHV-8, a DNA tumor virus that causes Kaposi's sarcoma, infects three types of dendritic cells: monocyte-derived dendritic cells, Langerhans cells, and interstitial dermal dendritic cells. We show that different receptors are used by this virus to infect these cells. DC-SIGN is a major receptor for infection of both monocyte-derived dendritic cells and interstitial dermal dendritic cells, yet the virus fully replicates only in the latter. HHV-8 uses langerin and the ephrin A2 receptor to infect Langerhans cells, which support full HHV-8 lytic replication. This infection of Langerhans cells and interstitial dermal dendritic cells results in an impaired ability to stimulate CD4^+^ helper T cell responses. Taken together, our data show that HHV-8 utilizes alternate receptors to differentially infect and replicate in these tissue-resident DC and support the hypothesis that these cells play an important role in HHV-8 infection and pathogenesis.

## INTRODUCTION

Kaposi's sarcoma (KS) is a rare tumor of the skin and mucous membranes, which can also involve visceral organs and lymph nodes. The etiological agent of this disease has been identified as human herpesvirus 8 (HHV-8), also known as KS-associated herpesvirus (KSHV) (1). In skin lesions, primarily spindle-shaped cells of endothelial origin form the tumor (2). However, these spindle cell tumors include myeloid cells of dendritic and monocytic lineages (3).

The skin and mucosa contain several types of dendritic cells (DC), particularly Langerhans cells (LC), which reside in the epidermis in close contact with keratinocytes and near the surface of the mucosa, and interstitial dermal DC (iDDC), which are resident in the dermis and mucosal layers (4, 5). LC and iDDC are professional antigen-presenting cells (APC) that take up and process cutaneous and mucosal antigens and migrate via the lymphatic circulation to draining lymph nodes, where they present the antigens to T and B cells. LC and iDDC express different pattern recognition receptors, C-type lectin receptors (CLR) and Toll-like receptors. LC express the CLR langerin (CD207) (6), while iDDC express DC-SIGN (CD209) (7) and mannose receptor (CD206) (8). Both langerin and DC-SIGN recognize pathogen-associated molecular patterns (PAMPs) and bind exclusively to specific carbohydrate moieties through their carbohydrate recognition domains (9, 10). The variable expression of these receptors on specific subsets of DC suggests that these cells can interact with the same antigens in different ways, with different outcomes.

We have previously shown that HHV-8 targets three types of APC through DC-SIGN, with divergent outcomes (11, 12). Thus, HHV-8 undergoes full-cycle replication in activated B cells, with the production of infectious virus (11, 13). In sharp contrast, the virus goes into latency after a limited production of lytic cycle proteins and little or no infectious virus in monocyte-derived DC (MDDC) and monocyte-derived macrophages (MDM) (12). This is similar to the nonproductive infection of endothelial cells by HHV-8 (12, 14). Given that classic KS is primarily a cutaneous neoplasia of the endothelial cell vasculature, the strategic position of LC and iDDC at skin and mucosal sites and the ability of these cells to capture pathogens suggest that these APC could become infected with HHV-8 and play an important role in the development of disease. In this regard, LC and iDDC are known to be important for certain virus infections, such as human immunodeficiency virus type 1 (HIV-1) (15), herpes simplex virus (16), and cytomegalovirus (CMV) (17) infections. There is, however, little information on infection of LC or iDDC by HHV-8. Indeed, systemic HHV-8 infection induces T cell responses that are not as robust as those generated against other persistent but controlled herpesvirus infections, i.e., Epstein-Barr virus (EBV) and CMV (17–20). One of the reasons why the HHV-8 T cell-specific response is more attenuated could be that APC infected with the virus fail to stimulate a vigorous T cell response. In fact, we previously reported that MDDC infected *in vitro* with HHV-8 show a decreased ability to induce memory T cell responses to recall antigens (12) and fail to produce interleukin 12 (IL-12) in response to maturation stimuli (21).

In the present study, we demonstrate that both LC and iDDC can be infected *in vitro* by HHV-8. Interestingly, contrary to what we observed with MDDC, both iDDC and LC support lytic viral replication. Furthermore, while HHV-8 uses DC-SIGN to infect iDDC, it uses both langerin and ephrin receptor A2 (EphA2) (22) to infect LC. Infected LC and iDDC also showed a decreased ability to prime naive CD4^+^ T cells. These data indicate that HHV-8 can target both LC and iDDC for productive infection and alter their function, supporting a role for these dermal and mucosal DC in HHV-8 infection and pathogenesis.

## RESULTS

### HHV-8 infects LC and iDDC.

**FIG 1:**
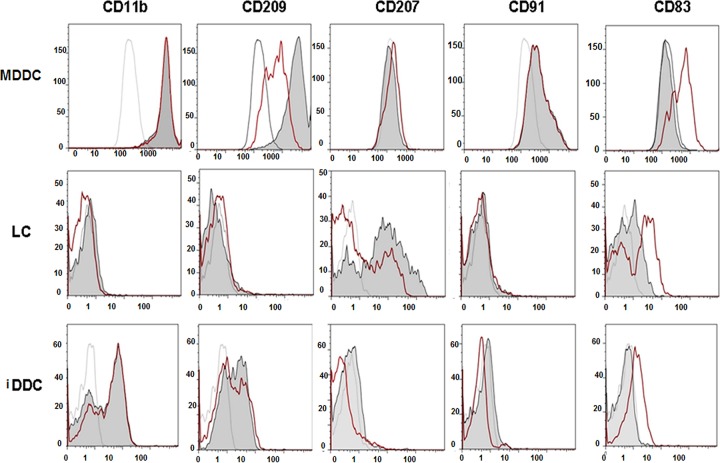
Phenotypes of cord blood-derived DC. MDDC, LC, and iDDC were derived from CD34^+^ neonatal cord blood cell precursors and stained with the listed MAbs to determine their phenotype. Empty histograms, isotype control; filled gray histograms, immature cells; red-line profiles, cytokine-matured cells (see Materials and Methods).

We previously showed the expression of HHV-8 lytic and latency cycle proteins in infected MDDC and MDM in the absence of productive virus infection (12). In this study, we determined if two types of tissue-resident DC, i.e., LC and iDDC, are susceptible to HHV-8 infection. To ascertain this, we first showed that immature LC and iDDC generated from CD34^+^ cells had distinctive phenotypic properties of these DC, as was previously reported (23). Thus, immature LC expressed langerin (CD207) and were DC-SIGN (CD209) negative (Fig. 1), as we previously reported (24). The generation of three phenotypically distinct and homogenous DC populations was further confirmed by the expression of the adhesion molecule CD11b and the scavenger receptor CD91 on iDDC and MDDC, but not on LC, as previously reported (23). Conversely, immature iDDC did not express CD207 but expressed CD209. A complete phenotypic characterization of the three distinct DC populations is shown in Fig. 1. The maturation of LC and iDDC was induced by using a cytokine-prostaglandin E_2_ (PGE_2_) cocktail (Fig. 1, red-line histograms) and was comparable to that of immature MDDC derived from CD34^−^ CD14^+^ cells of the same cord blood (12). Although no expression of MDDC- or iDDC-specific markers was detected in the LC cultures, and to ensure the most homogeneous population, immature LC were further purified by CD1a magnetic bead separation (see Fig. S1 in the supplemental material). As shown, CD1a^+^ cell purification further increases the percentages of cells expressing langerin (CD207) in culture while maintaining the expression of HLA-I, HLA-II, CD83, and CD86.

**FIG 2:**
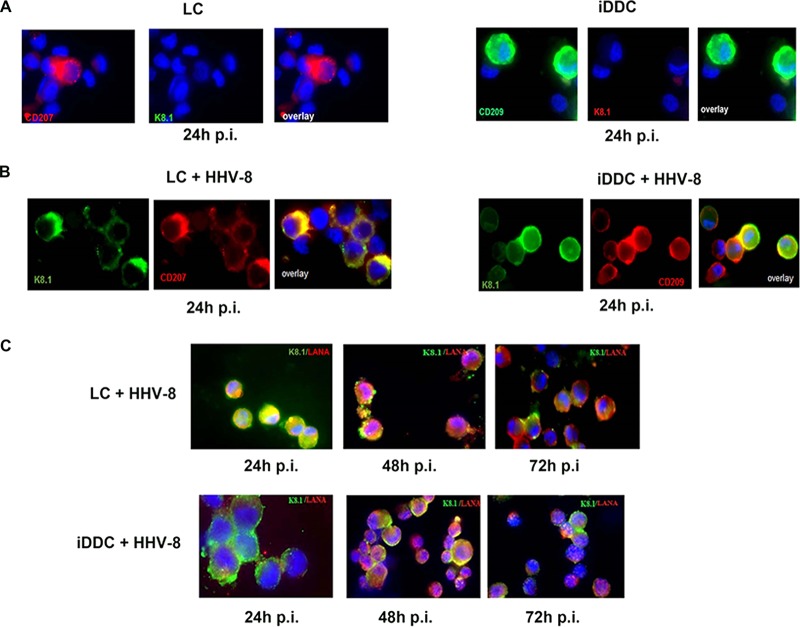
HHV-8 infects and replicates in LC and iDDC. (A) Uninfected, control cells. (B). Cells were infected with HHV-8 and stained with an anti-DC-SIGN (iDDC) or antilangerin (DC207) (LC) MAb and anti-K8.1A/B at 24 hpi. (C) Infected LC or iDDC were stained with anti-K8.1A/B or anti-LANA MAb at 24, 48, and 72 hpi, as described in Materials and Methods (magnification, ×60). Blue, DAPI (4′,6-diamidino-2-phenylindole) nuclear stain.

We next assessed HHV-8 infection of these cells by analysis of viral lytic cycle protein expression. Control, uninfected cells are shown in Fig. 2A. Infection of cultured LC and iDDC resulted in the expression of the lytic cycle protein K8.1 within 24 h of infection (Fig. 2B). As shown, HHV-8-infected iDDC expressed DC-SIGN (CD209), and LC expressed langerin (CD207) (Fig. 2B). We conclude that the expression of C-type lectin receptors is sufficient to support infection of LC and iDDC by HHV-8.

We previously observed that MDDC infected with HHV-8 express lytic protein for 24 to 48 h; by 72 h postinfection, we could detect the expression of the latency protein LANA only (12). Interestingly, in the present study, by 72 h after infection of both LC and iDDC, there was a mixture of cells expressing either viral lytic or latency proteins, suggesting that a proportion of these cells was still undergoing viral lytic replication (Fig. 2C). Lytic replication in these cells was not reflected by a significant decrease in cell viability or cell numbers in either type of HHV-8-infected DC culture (Fig. S2). As shown, in some instances, we found both nuclear and cytoplasmic expression of LANA. This unusual pattern of LANA expression has also been reported by others, and it has been proposed that these cytoplasmic isoforms may represent a mechanism of promoting reactivation from latency (25).

### Role of DC-SIGN and langerin in HHV-8 infection of iDDC and LC.

**FIG 3:**
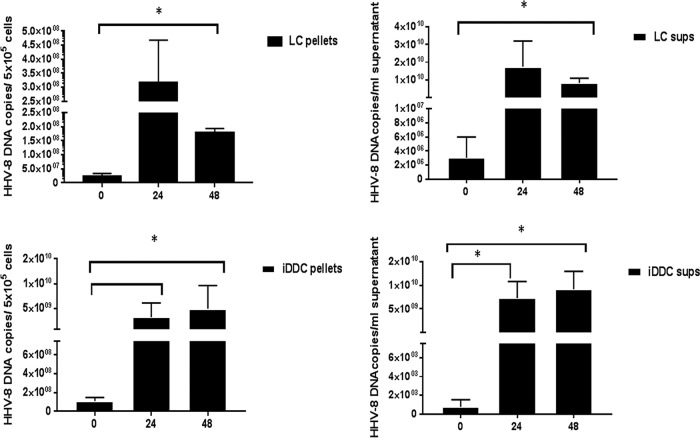
HHV-8 replicates in LC and iDDC. Shown are quantitative DNA results from a real-time PCR assay of total DNA that was extracted from infected LC and iDDC and cell culture supernatants (sups) and treated with DNase prior to an RT-PCR assay (see Materials and Methods). Levels of viral DNA were significantly higher in infected LC and iDDC between 0 h and 48 h postinfection in both cell pellets and culture supernatants. Data represent means ± standard errors (*n* = 4). *, *P* ≤ 0.05.

Next, we investigated if these APC support productive viral replication, as determined by cell-associated and extracellular levels of viral DNA in HHV-8-infected iDDC and LC cultures. Assessment of infected iDDC and LC from 4 independent determinations shows that after the first 24 h of infection, there were increases in the levels of HHV-8 DNA in both cell pellets and supernatants compared to the levels in samples tested at 0 h (i.e., cells or supernatants sampled after washing and replacement of culture medium with fresh medium) (Fig. 3). In iDDC, we detected significant increases (*P* < 0.05) in the levels of viral DNA after 24 h and 48 h of infection in both cell pellets and culture supernatants. We also observed a significant increase in the level of HHV-8 DNA in LC culture supernatants at between 0 h and 48 h of infection (*P* < 0.05). In contrast, there was no increase in viral DNA levels in infected MDDC, as we previously reported (12) (Fig. S3).

**FIG 4:**
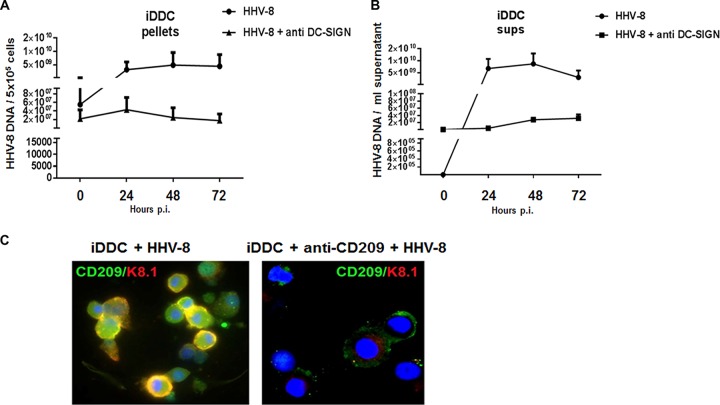
Infection of iDDC with HHV-8 is blocked by anti-DC-SIGN MAb. (A and B) iDDC were left untreated or treated with an anti-DC-SIGN MAb prior to infection with HHV-8. Viral DNA amounts were determined as described in the legend to Fig. 3. (A) Cell pellets; (B) cell culture supernatants (sups). Shown are means ± standard errors (*n* = 5). DNA levels measured at time zero postinfection reflected residual virus bound to cells after incubation and extensive washing. (C) Immunofluorescence of untreated or anti-DC-SIGN-treated iDDC infected with HHV-8 for 24 h. Cells were stained with anti-DC-SIGN (green) and anti K8.1A/B (red). Blue, DAPI nuclear stain. Data are representative of results from 5 independent experiments.

We previously demonstrated that DC-SIGN expressed on MDDC serves as a receptor for HHV-8 infection (12). iDDC and LC express type II transmembrane C-type lectin receptors, DC-SIGN and langerin, respectively. Therefore, we set out to determine if these molecules could serve as a portal for infection of these DC. iDDC were infected in the presence of an anti-DC-SIGN monoclonal antibody (MAb), and the levels of HHV-8 DNA in cell pellets and cell culture supernatants were measured over time. Cells were also stained with an anti-K8.1A/B MAb to measure the expression of viral proteins. As shown in Fig. 4A, viral DNA in the anti-DC-SIGN MAb-treated, infected cell cultures was maintained at baseline levels for up to 72 h postinfection, while viral DNA levels were increased in untreated, infected iDDC cultures.

HHV-8 was maintained in the supernatant of cells infected in the presence of DC-SIGN-treated cells at the same levels throughout the 3 days of infection. However, an increase in the HHV-8 DNA level was detected in the supernatants of infected iDDC (Fig. 4B). Pretreatment of iDDC with the anti-DC-SIGN MAb resulted in a sharp decrease in the percentage of infected cells, as measured by an immunofluorescence assay (IFA); i.e., <5% of cells in the anti-DC-SIGN-pretreated, HHV-8-infected cultures were positive for K8.1 protein expression, compared to 44% of cells in the untreated, infected cultures (Fig. 4C). Baseline was defined as the number of HHV-8 DNA copies from cell pellets after virus incubation and washing. The DNA copies detected at 0 h in the cell pellets most likely reflect residual virus nonspecifically bound to the cell membrane. The baseline for the cell culture supernatant was measured after virus incubation, washing, and replenishing the cultures with fresh culture medium. Virus DNA was detected at 0 h in the supernatants of anti-DC-SIGN-treated cultures, in contrast to the low HHV-8 DNA levels detected in the infected, untreated cultures. We hypothesize that in the blocked cultures, virus that nonspecifically bound to the cell surface was released and became detectable in the cell culture supernatants. In fact, the amount of viral DNA in such cultures stays constant over time, as opposed to the increases in virus DNA levels released into the supernatants of untreated cells.

**FIG 5:**
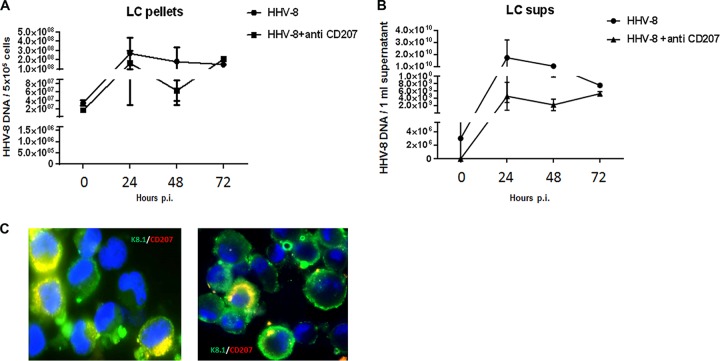
HHV-8 infection of LC is partially blocked by antilangerin MAb. (A and B) LC were left untreated or treated with an antilangerin MAb (anti-CD207) prior to infection with HHV-8. Viral DNA amounts were determined as described in the legend to Fig. 3. (A) Cell pellets; (B) cell culture supernatants. Shown are means ± standard errors (*n* = 5). (C) Immunofluorescence of untreated (left) or antilangerin (CD207)-treated (right) LC infected with HHV-8 for 24 h. Cells were stained with anti-CD207 (red) and anti-K8.1A/B (green). Data are representative of results from 5 independent experiments.

Since langerin is a receptor for pathogens such as HIV-1 (15), we set out to determine if infection of LC by HHV-8 could involve langerin. We infected LC with HHV-8 in the presence of anti-langerin MAb and measured viral DNA and protein expression levels. As shown in Fig. 5A, treatment of LC with an anti-langerin MAb derived from clone DCGM4 (Beckman Coulter), used previously by de Witte et al. (15) to inhibit HIV-1 infection of LC, did not result in significant decreases in the levels of HHV-8 DNA in either the cell pellets or culture supernatants. Baseline values were determined as described above for the iDDC cultures. Furthermore, after treatment, we still detected LC expressing the viral protein K8.1 but not langerin by an IFA, confirming that the blocking of this receptor did not prevent infection as efficiently as did the blocking of DC-SIGN on iDDC (Fig. 5B). The lack of detection of langerin by IFAs (Fig. 5C) confirmed the efficiency of blocking the expression of this receptor. Similar results were obtained by using a different anti-langerin MAb (clone 343828; R&D Systems) (Fig. S4). Treatment of either iDDC or LC with anti-mouse IgG as a control in the MAb blocking experiments did not interfere with virus infection, as measured by the number of viral DNA copies (Fig. S5).

**FIG 6:**
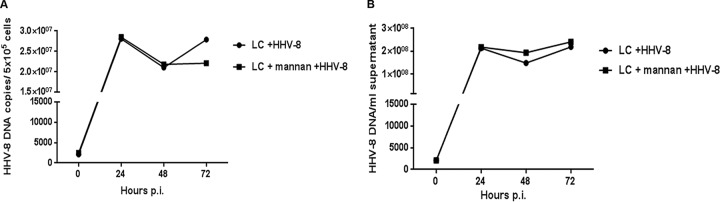
HHV-8 infection of LC is not inhibited by mannan. LC were left untreated or treated with mannan prior to infection with HHV-8. Viral DNA amounts were determined as described in the legend to Fig. 3. (A) Cell pellets; (B) cell culture supernatants. Data are representative of results from 2 independent experiments.

**FIG 7:**
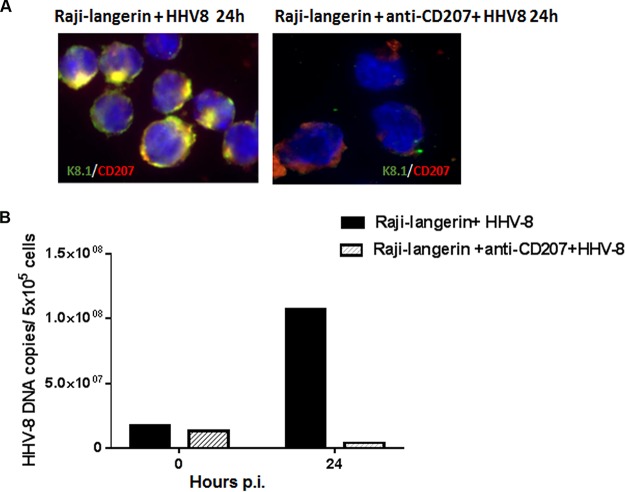
Langerin expression renders resistant cells susceptible to HHV-8 infection. (A) Raji-langerin cells were left untreated (left) or treated with an antilangerin (CD207) MAb (right) prior to infection with HHV-8. (B) Viral DNA levels in untreated or antilangerin (CD207) MAb-treated cultures were determined as described in the legend to Fig. 3. Data are representative of results from 2 independent experiments.

To exclude the possibility that the level of HHV-8 infection that we observed in the langerin-blocked LC cultures could be due to contaminating, DC-SIGN-expressing iDDC, we treated LC with mannan (CD206) prior to infection. Mannan is a natural ligand for DC-SIGN, and LC do not express the mannan receptor (8). As shown in Fig. 6, pretreatment with mannan did not interfere with infection of LC, as measured by viral DNA levels in both cell pellets (Fig. 6A) and cell culture supernatants (Fig. 6B). Furthermore, this also confirms the homogeneity of our cultures. To better define whether langerin is a receptor for HHV-8 and if the blocking MAb used was effective, we infected Raji cells that we previously transfected to express langerin (Raji-langerin) (15). We showed previously that Raji cells are refractory to infection with HHV-8 but become permissive when they are transfected with DC-SIGN (26). As shown in Fig. 7, Raji-langerin cells can also be productively infected by HHV-8, as measured by both IFAs and the levels of viral DNA (Fig. 7A and B, respectively). Infection could be completely abrogated by pretreatment with an antilangerin MAb, suggesting that langerin can mediate infection by HHV-8 and that this can be effectively blocked by the antilangerin MAb used in our study. These data indicate that both LC and Raji-langerin cells support productive HHV-8 infection and replication. The lack of inhibition of HHV-8 infection in LC but not Raji-langerin cells by the antilangerin MAb suggests that multiple receptors are used for infection of LC by this virus.

**FIG 8:**
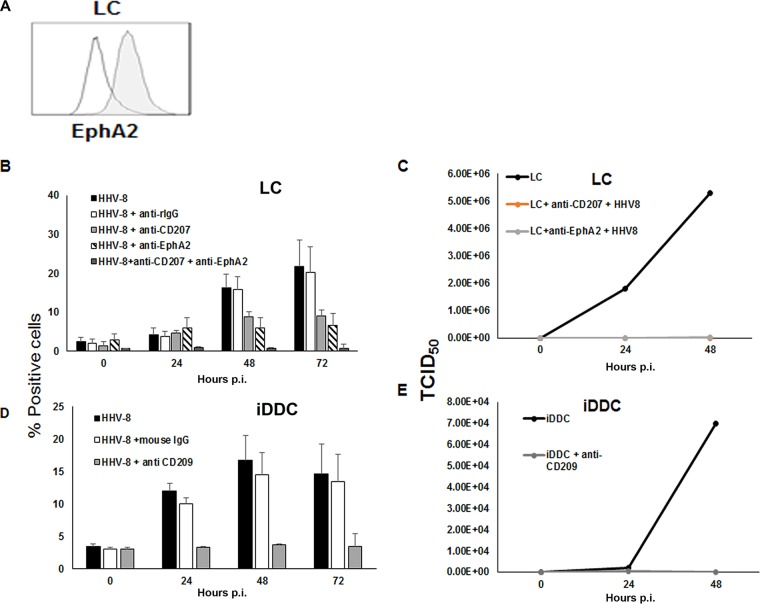
Infection of LC is blocked by anti-EphA2 polyclonal Ab. (A) LC express the EphA2 receptor. Empty histograms, isotype control; filled gray histograms, EphA2. (B) LC were left untreated or incubated with anti-EphA2 polyclonal antibody for 1 h at room temperature and then infected with HHV-8 expressing GFP for up to 72 h. Alternatively, cells were treated with anti-CD207 MAb or with the two Abs combined, as described in Materials and Methods. Cells treated with rabbit polyclonal IgG (rIgG) were used as a control. Cells were sampled at the indicated time points, and levels of GFP expression were measured by flow cytometry. (C) Cell culture supernatants of untreated or blocked HHV-8-infected cultures were collected and tested to determine the TCID_50_ as described in Materials and Methods. (D) iDDC were left untreated or incubated with an anti-DC-SIGN MAb or mouse IgG and then infected with HHV-8 expressing GFP for up to 72 h. (E) Supernatants from infected or blocked iDDC cultures were collected and tested to determine the TCID_50_ as described in Materials and Methods. Data were acquired with Diva and analyzed with FlowJo software. Wells were scored positive based on a cutoff set by the averages of data from 6 uninfected supernatants ± 3 SD. The TCID_50_ was then calculated by using the Reed-Muench calculator (39).

EphA2 has been described as a receptor for HHV-8 (22). This receptor has been shown to be expressed by Langerhans-like DC *in vitro* (27). Indeed, we also detected the expression of this receptor on LC generated in our culture system (Fig. 8 A). Therefore, we investigated whether blocking of EphA2 on LC prevented HHV-8 infection. For this set of experiments, we used a clone of recombinant HHV-8 strain rKSHV.152 (28) designated BAC16, and infection was determined by flow cytometry. This virus expresses recombinant green fluorescent protein (GFP) upon infection of target cells. LC were infected at a multiplicity of infection (MOI) of 0.5, as determined by titration in K562-DC-SIGN cells (not shown). Parallel cultures were blocked with either a polyclonal anti-EphA2 antibody (Ab), as previously described (22); the antilangerin MAb; or both prior to infection with the recombinant virus. Cells were incubated with the virus preparation for 2 h at 37°C and washed, and fresh medium was added. Cells were further incubated for up to 72 h, and samples were taken at the 24-, 48-, and 72-h time points to determine the percentage of cells expressing GFP by fluorescence-activated cell sorter (FACS) analysis. As shown in Fig. 8B, blocking of EphA2 inhibited productive infection of LC by 70.1% by 72 h postinfection, while blocking with the antilangerin MAb or the combination of the polyclonal anti-EphA2 Ab and antilangerin MAb inhibited infection by 58% and 99.2%, respectively. We also measured the HHV-8 50% tissue culture infective dose (TCID_50_) in supernatants from infected, untreated LC cultures and cultures treated with the antibody blocking reagents. As shown in Fig. 8C, blocking with either antibody or their combination completely inhibited the recovery of infectious virus in the cell culture supernatants.

This finding was seemingly in contrast to data shown in Fig. 8B, where virus is still detectable in a proportion of infected, blocked cultures, particularly at earlier time points. It needs to be noted that the TCID_50_ assay measures the amount of free, infectious virus in the cell culture supernatants, and therefore, the effect of blocking is more evident in this setting. The data shown in Fig. 8C may also seem to be in contrast with the data shown in Fig. 5B, where virus DNA was still detectable in the blocked cell culture supernatants, due to the greater sensitivity of virus DNA real-time PCR (RT-PCR) than of the TCID_50_ assay. We also measured infection of iDDC and the effect of blocking with the DC-SIGN MAb by using the same methods. As shown in Fig. 8D, blocking with the DC-SIGN MAb reduced the percentage of infected cells by 77% by 72 h postinfection and completely abrogated the recovery of infectious virus in the cell culture supernatant (Fig. 8E).

### HHV-8 infection and DC marker expression.

**FIG 9:**
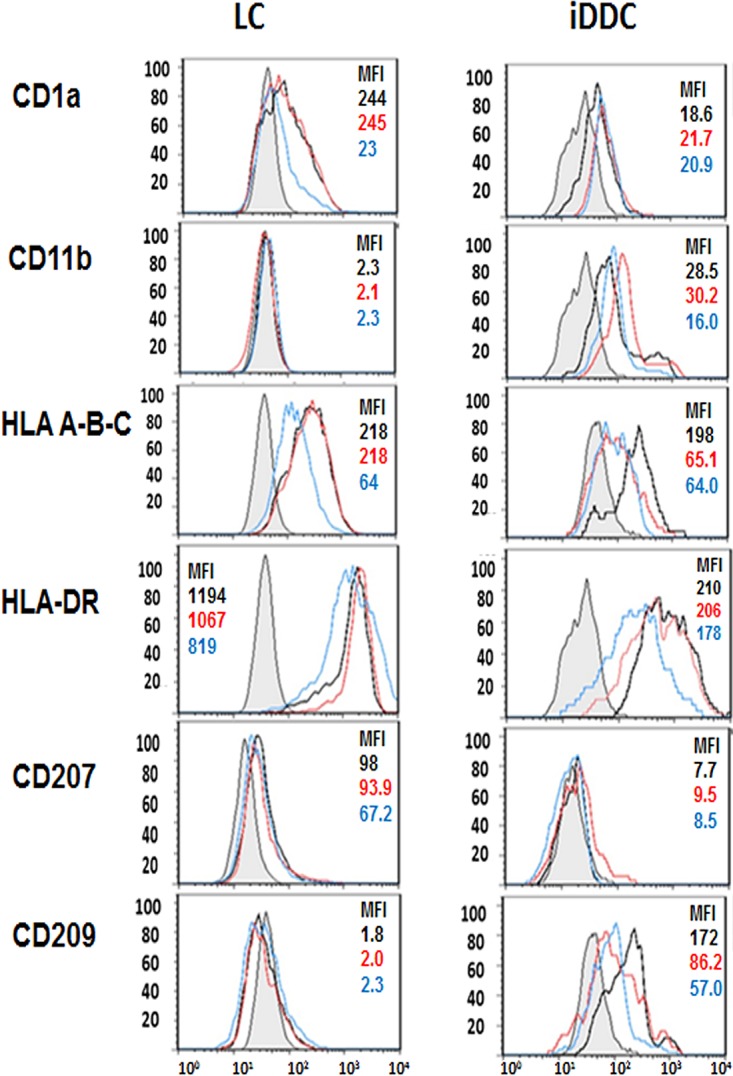
Effect of HHV-8 infection on cell surface phenotypes of LC and iDDC. LC and iDDC were left untreated (black-line profile) or infected with HHV-8 for 24 h (red-line profile) and 48 h (blue-line profile). Cells were collected, stained with the listed MAbs, and read on an LSRII flow cytometer. Gray histograms, isotype controls. MFI values at 0, 24, and 48 hpi are shown.

We next examined if infection with HHV-8 influences the expression of cell markers important for the antigen-presenting functions of iDDC and LC. As shown in Fig. 9, both LC and iDDC exhibited downregulation of major histocompatibility complex (MHC) class I expression at 24 and 48 h postinfection, while the expression of MHC class II (HLA-DR), CD1a, and CD11b was not altered at 24 h of infection but showed a slight downregulation at 48 h in LC, as indicated by the mean fluorescence intensity (MFI). The ability of HHV-8 to downregulate the expression of MHC class I was previously noted (29, 30) and is considered one of several immune escape strategies employed by this herpesvirus. As reported previously by others (31), we observed the downregulation of CD1a expression in HHV-8-infected LC. However, this effect was not observed in infected iDDC. HHV-8-infected iDDC also showed a downregulation of DC-SIGN (CD209) expression, as we previously reported for infected MDDC (12), supporting that this molecule acts as a receptor for HHV-8 in iDDC. In contrast, we did not detect any changes in the expression of CD207 in infected LC.

### Effect of HHV-8 infection on T cell priming by LC and iDDC.

**FIG 10:**
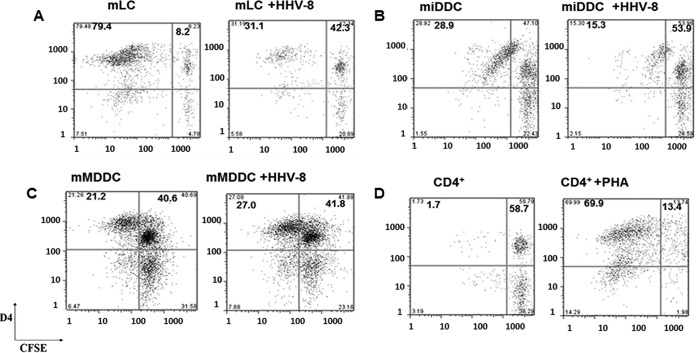
Proliferation of neonatal CD4^+^ T cells in response to allogeneic stimulation with HHV-8-infected or uninfected mature MDDC, iDDC, and LC (A to C). Uninfected or HHV-8-infected mature LC (mLC) (A), mature iDDC (B), or mature MDDC (C) were mixed with allogeneic CD4^+^ T cells labeled with CFSE and cultured for 1 week. (D) T cells stimulated with PHA (2 μg/ml) were used as controls. Proliferation was measured as a dilution of CFSE stain by flow cytometry.

LC have been described as potent stimulators of allogeneic, naive CD4^+^ T cells, inducing their differentiation into either gamma interferon (IFN-γ)-secreting Th1 cells or Th2 effectors secreting IL-4, IL-5, and IL-13 (8, 32). We therefore examined if infection of LC with HHV-8 affects their ability to induce naive CD4^+^ T cell proliferation in an allogeneic mixed-lymphocyte reaction (allo-MLR). As shown in Fig. 10, HHV-8-infected mature LC were poor inducers of CD4^+^ T cell proliferation compared to uninfected LC. Infected iDDC also showed reduced allostimulatory activity although not as strongly as infected LC (Fig. 10B). In contrast, HHV-8-infected MDDC maintained this T cell-priming function (Fig. 10C). CD4^+^ T cells stimulated with phytohemagglutinin (PHA) were used as a control (Fig. 10D). Cell viability was determined by trypan blue exclusion. We did not observe a significant difference between the viability of HHV-8-infected cells and that of uninfected cells, consistent with the data shown in Fig. S3 in the supplemental material.

## DISCUSSION

These results show that HHV-8 infects and replicates in LC and iDDC *in vitro*, with the production of infectious virus as determined by DNA RT-PCR and TCID_50_ assays and the expression of lytic and latency cycle viral proteins. We analyzed HHV-8 infection in these cells, which express two different C-type lectins (DC-SIGN and langerin), under the hypothesis that they would follow the pattern of viral infection that we previously observed for MDDC, i.e., limited lytic viral replication that required DC-SIGN expression (12). In the case of HIV-1 infection, langerin has been described to capture the virus and transport it to Birbeck granules for degradation, thus forming a protective barrier to HIV-1 dissemination (15). We hypothesized that a similar mechanism could be in place for HHV-8 infection. Surprisingly, we detected HHV-8 replication in both LC and iDDC, with the expression of lytic proteins during the first 48 h in culture; by 72 h, the cultures displayed a mixture of cells expressing either latency or lytic proteins. Viral replication was further demonstrated by an increase in the level of viral DNA detected in the cell culture pellets and supernatants and the production of infectious virus. Importantly, we found that langerin was not unequivocally required for HHV-8 infection of LC in that antilangerin MAb blocked only 48% of HHV-8 infection of LC. LC did not express DC-SIGN, a known receptor for HHV-8 on MDDC (11, 12). LC also did not express mannose receptor, and treatment of LC with mannan, a natural ligand for DC-SIGN and mannose receptor, had no effect on blocking HHV-8 infection of these APC. Thus, DC-SIGN and mannose receptor did not account for the HHV-8 replication observed in LC.

Interestingly, Raji cells transfected to express langerin (Raji-langerin) supported full lytic cycle replication of HHV-8 compared to wild-type (wt) Raji cells, which were refractory to HHV-8 infection. We have previously shown that Raji cells can be infected with HHV-8 only if they have been transfected to express DC-SIGN (11). Moreover, blocking with a antilangerin MAb inhibited HHV-8 infection of Raji-langerin cells, as measured by decreased levels of viral DNA and expression of viral proteins. This supports that langerin is involved in HHV-8 infection, possibly as a receptor for the virus. Because of the apparent discrepancy between the effects of langerin blocking on LC and Raji-langerin cells, we investigated if another receptor for HHV-8, EphA2, could act as an alternative receptor for HHV-8 on LC. In fact, blocking of EphA2 on LC with a polyclonal Ab inhibited 70.1% of HHV-8 infection in these cells. Moreover, blocking of both EphA2 and langerin with a combination of their respective Abs inhibited over 98% of HHV-8 replication in LC. These results support that HHV-8 can use more than one receptor to infect LC, i.e., langerin and EphA2.

Infected LC did not show an alteration in langerin expression but displayed decreased expression levels of MHC class I and CD1a at 48 h postinfection. MHC class I is known to be downregulated after HHV-8 infection (29, 30, 33), and CD1a downregulation was also previously found in infected cells (31). Interestingly, iDDC supported productive, lytic infection with HHV-8, in contrast to our previous finding that the virus undergoes minimal replication in MDDC (12). Infection of iDDC with HHV-8 induced the downregulation of DC-SIGN, and treatment with an anti-DC-SIGN MAb inhibited productive infection in these cells, confirming that DC-SIGN is a major receptor for HHV-8 in both iDDC and MDDC. It is not clear why iDDC sustain productive infection by HHV-8, in contrast to MDDC. These DC, while similar in their expression levels of DC-SIGN, have distinct differences, including the expression of CD14 on iDDC but not on MDDC. Moreover, preliminary data indicate that natural, skin-derived iDDC can also be productively infected by HHV-8 (our unpublished data). Further studies are needed to define the replicative pathways of HHV-8 in these different DC.

It has been proposed that LC preferentially activate cell-mediated immunity and are strong activators and primers of CD4^+^ T cell responses to antigens (32). In contrast, iDDC are more important in the development of humoral immunity by inducing the generation of follicular helper T cells that are able to induce naive B cells to switch isotypes and produce antibodies. In our study, HHV-8-infected LC and iDDC had a diminished capacity to stimulate allogeneic CD4^+^ T cells in an allo-MLR. These results also support a role for EphA2 in HHV-8 infection of LC; that is, the binding and activation of this kinase induce tyrosine phosphorylation, which in turn activates mitogen-activated protein kinase (MAPK). MAPK activation influences the activation and maturation of DC by affecting integrin affinity and the cytoskeleton (27). Thus, the interaction of HHV-8 with EphA2 on LC could in part be why infected LC showed a reduced ability to stimulate CD4^+^ T cells. On the other hand, infected MDDC did not have an impaired ability to stimulate naive CD4^+^ T cells to proliferate. This could be due to the lack of HHV-8 replication in MDDC compared to LC and iDDC. In fact, it has been reported that in the case of measles virus (MV) infection of dermal DC and LC, the loss of DC stimulatory functions is related to virus replication, since UV-treated supernatants of MV-infected DC do not alter the allostimulatory capacity of DC (34).

Taken together, our data indicate that HHV-8 differentially infects and replicates in several types of primary myeloid DC and support the role of these cells in HHV-8 infection and pathogenesis. Combined with our previously reported results (12), it is evident that HHV-8 can undergo limited, nonproductive infection of myeloid-origin DC (i.e., MDDC) and macrophages (i.e., IL-13-activated MDM) while productively infecting LC and iDDC. Lytic infection of these myeloid cells with HHV-8 is restricted by the expression of the DC-SIGN receptor on MDDC, iDDC, and IL-13-activated MDM and langerin and EphA2 receptors on LC. Interestingly, we have also shown that both DC-SIGN expression and cell activation are required for full-cycle lytic replication of HHV-8 in B lymphocytes of blood and tonsils (12, 13). Given the well-documented dependence of HHV-8 lytic replication in experimental cell models on transcription factors or transcriptional coactivators (35, 36), we speculate that such cell activation factors contribute to the permissiveness of primary myeloid DC and macrophages, as well as B lymphocytes, to HHV-8 lytic replication.

## MATERIALS AND METHODS

### Generation of MDDC, LC, and iDDC.

Approval was obtained from the University of Pittsburgh institutional review board prior to these studies involving human subjects. To obtain immature MDDC, CD14^+^ monocytes were positively selected from neonatal cord blood (Magee-Women's Hospital, Pittsburgh, PA) by using anti-CD14 MAb-coated magnetic beads (Miltenyi Biotec, Auburn, CA) to a purity of >95% viable cells (trypan blue dye exclusion assay) (24). MDDC were derived by culture of purified neonatal cord blood monocytes with 1,000 U/ml recombinant granulocyte-macrophage colony-stimulating factor (GM-CSF) (Amgen, Thousand Oaks, CA) and 1,000 U/ml recombinant IL-4 (R&D Systems, Minneapolis, MN). Immature MDDC were treated on day 5 with CD40L (1 μg/ml; Alexis, San Diego, CA) or a combination of inflammatory cytokines and PGE_2_, i.e., IL-1β (2 ng/ml; R&D Systems), IL-6 (1,000 IU/ml; R&D Systems), tumor necrosis factor alpha (TNF-α) (10 ng/ml; R&D Systems), and PGE_2_ (5 μM; Pfizer, Vienna, Austria), for 40 h to induce DC maturation (37).

To obtain LC and iDDC, mononuclear cells from neonatal cord blood samples were treated with anti-CD34 MAb-coated magnetic microbeads (Miltenyi Biotec, Auburn, CA) to isolate CD34^+^ stem cells at a purity of >98% (23, 24). CD34^+^ cells were cultured in X-Vivo 15 medium (BioWhittaker, Walkersville, MD) to generate LC or in complete Iscove's modified Dulbecco's medium (Invitrogen, Carlsbad, CA) supplemented with 20% heat-inactivated, human AB^+^ serum (Sigma, St. Louis, MO) to generate iDDC. The culture medium for both LC and iDDC was supplemented with GM-CSF (1,000 IU/ml), TNF-α (5 ng/ml), c-*kit* ligand (20 ng/ml; R&D Systems), and Fm-related tyrosine kinase 3 (FLT-3) ligand (50 ng/ml; R&D Systems). Cultures were replenished with cytokines and media on day 3. On day 5, the cells were washed and cultured with the same cytokines except without the c-*kit* ligand and the FLT-3 ligand. All cultures were transferred into X-Vivo 15 medium. Cytokines and medium were replenished every other day for the duration of the culture. For the development of LC, transforming growth factor β1 (TGF-β1) (10 ng/ml; R&D Systems) was added throughout the culture period. To ensure that the most homogeneous population of LC was used in the infectivity and functionality experiments, we further purified LC from cultures by anti-CD1a MAb magnetic bead separation. Briefly, anti-CD1a beads (Miltenyi Biotec) were incubated with LC cultures according to the manufacturer's protocol. Cells eluted from the positive and negative fractions were characterized by flow cytometry for LC markers. For the generation of iDDC, IL-4 (500 IU/ml) was added on day 5 with GM-CSF and TNF-α. To induce maturation, both LC and iDDC were treated with a cytokine-PGE_2_ mixture for 40 h (37). At the end of the culture period, the cell phenotype was determined by flow cytometry.

### Flow cytometry.

The expression of cell surface molecules was determined by flow cytometry (Fortessa; Becton Dickinson) using a fluorescein isothiocyanate (FITC)- or phycoerythrin (PE)-conjugated MAb specific for HLA-DR, CD1a, CD11b, CD14, CD91, CD80, CD86, CD83, CD209 (DC-SIGN), or CD207 (langerin) or polyclonal anti-EphA2 (Santa Cruz Biotechnology) for 30 min at 4°C, and the cells were then fixed with 1% paraformaldehyde. Cells stained with isotype immunoglobulin served as controls. Optimal photomultiplying tube (PMT) voltage settings for each fluorescence channel were determined on the day of the experiment by using cytometer setup and tracking (CST) beads (Standardization and Tracking beads; BD Biosciences). These voltage values are set to give the lowest coefficient of variation (CV) for that individual fluorescence channel.

### Immunofluorescence microscopy.

Immunofluorescence microscopy was done on HHV-8-infected or uninfected cells by using an anti-mouse MAb directed against ORF-K8.1A/B (ABI) as previously described (12). Briefly, cells were counted, spotted onto poly-l-lysine-coated slides, fixed in 4% paraformaldehyde for 20 min, and permeabilized with buffer for 20 min at room temperature. Cells were incubated with the K8.1A/B MAb directly conjugated with phycoerythrin or Texas Red by using a Zenon labeling kit (Molecular Probes). Cells were also stained by direct immunofluorescence with an FITC-conjugated anti-CD209 MAb (clone 120507; R&D Systems) or anti-CD207 (clone DCGM4 or clone 343828). To avoid nonspecific binding of IgG, a blocking step was added by using SuperBlock blocking buffer (Pierce). Uninfected cells and cells stained with isotype controls were used as controls. Cells were visualized at a ×60 magnification with a Nikon Eclipse E600 microscope.

### Virus purification and infection of cells.

Concentrated supernatants and cell-free lysates from 2 × 10^9^ 12-*O*-tetradecanoylphorbol-13-acetate (TPA; Sigma)-induced BCBL-1 cells were used as the source of HHV-8 and purified as previously described (12). In some experiments, cells were infected with the BAC16 clone of rKSHV.152 (28). In this case, infected cells were identified by GFP expression and enumerated by flow cytometry. For wild-type virus, titers were defined as the number of encapsidated (DNase-resistant) DNA genomes, as measured by RT-PCR. An average of 1 × 10^7^ copies of viral genomes were used to infect 1 × 10^6^ cells for 2 h at 37°C, and the cells were washed and incubated for up to 3 days in cell culture medium at 37°C in 5% CO_2_. For receptor-blocking studies, cells were pretreated with 20 μg/ml of an anti-human CD209 MAb (clone 120507; R&D Systems) or anti-CD207 MAb (clone DCGM4; R&D Systems) or with polyclonal anti-EphA2 Ab (1 mg/ml; Santa Cruz Biotechnology). Cells incubated with 20 μg/ml of mouse IgG (Sigma) or 1 mg/ml rabbit polyclonal IgG (Sigma) were used as controls. In some experiments, cells were incubated with 100 μg/ml of mannan (Sigma) for 1 h at 4°C before exposure to HHV-8.

### TCID_50_ assay.

The TCID_50_ assay was modified from our previously reported methods (38). Briefly, K562 cells transfected to express CD209 (12) were seeded into a 96-well plate and incubated with supernatants from rKSHV.152 BAC16-infected cell cultures (28). Each supernatant was serially 10-fold diluted from 10^−1^ to 10^−6^ and tested in 6 replicate wells. Supernatants from uninfected parallel cultures were used as negative controls. Plates were read on a Fortessa flow cytometer, and data were analyzed with FlowJo v10 software. Wells were scored positive based on a cutoff set by the average values for 6 uninfected supernatants ± 3 standard deviations (SD). The TCID_50_ was then calculated by using the Reed-Muench calculator (39).

### Quantitative PCR assay for HHV-8.

HHV-8 DNA levels were measured by a quantitative, real-time TaqMan PCR assay with a primer set specific for the HHV-8 alkaline exonuclease gene (open reading frame 37 [ORF37]), as previously described (12). Standardization of HHV-8 DNA quantitation was done by using known quantities of HHV-8 DNA (ABI). To determine viral DNA levels in infected cells, cellular DNA was extracted by using the QIAamp blood DNA miniprep kit (Qiagen). To determine viral DNA levels from encapsidated virus in cell culture supernatants, the experimental samples were pelleted by ultracentrifugation and treated with 1 μl DNase I (Invitrogen), followed by phenol-chloroform extraction. Cell-free supernatants were centrifuged at 22,000 rpm for 60 min at 4°C to pellet the virus and then treated with DNase as described above prior to RT-PCR assays.

### Mixed-lymphocyte reaction.

To assess the primary T cell-stimulatory capacities of LC and iDDC, neonatal allogeneic T cells (1 × 10^5^ cells/well) were labeled with carboxyfluorescein succinimidyl ester (CFSE) according to the manufacturer's instructions (Sigma) and stimulated in a 96-well plate with HHV-8-infected or uninfected matured LC (1 × 10^4^ cells/well). Cells were harvested after 7 days, and cell proliferation was assessed by measuring the CFSE fluorescence per cell. Cells were stained with anti-CD3, anti-CD4, and anti-CD8 MAbs to differentiate the proliferation of CD4^+^ and CD8^+^ T cells.

## Supplementary Material

Supplemental material
