# Supplementary material for: Human Herpesvirus 8 Infects and Replicates in Langerhans Cells and Interstitial Dermal Dendritic Cells and Impairs Their Function
Source: J Virol. 2017 Sep 27;91(20):e00909-17. doi: 10.1128/JVI.00909-17 (PMC5625489; doi:10.1128/JVI.00909-17)
Supplement: Supplemental material [file supp_91_20_e00909-17__index.html]

Supplemental material 

# Human Herpesvirus 8 Infects and Replicates in Langerhans Cells and Interstitial Dermal Dendritic Cells and Impairs Their Function

## Supplemental material

- Supplemental file 1 -

  Fig. S1 (Phenotypic characterization of CD1a+-sorted LC.)

  Fig. S2 (Cell counts and viability in infected iDDC and LC.)

  Fig. S3 (HHV-8 does not replicate in neonatal MDDC.)

  Fig. S4 (HHV-8 infection of LC is partially blocked by anti-CD207 mAb.)

  Fig. S5 (HHV-8 infection of iDDC is blocked by anti-CD209 mAb, but not by anti-CD207, in Langerhans cells.)

  PDF, 572K
