# Supplementary material for: Human Herpesvirus 8 Infects and Replicates in Langerhans Cells and Interstitial Dermal Dendritic Cells and Impairs Their Function
Source: J Virol. 2017 Sep 27;91(20):e00909-17. doi: 10.1128/JVI.00909-17 (PMC5625489; doi:10.1128/JVI.00909-17)
Supplement: Supplemental material [file JVI.00909-17_zjv999182977s1.pdf]

**Fig.S 1**

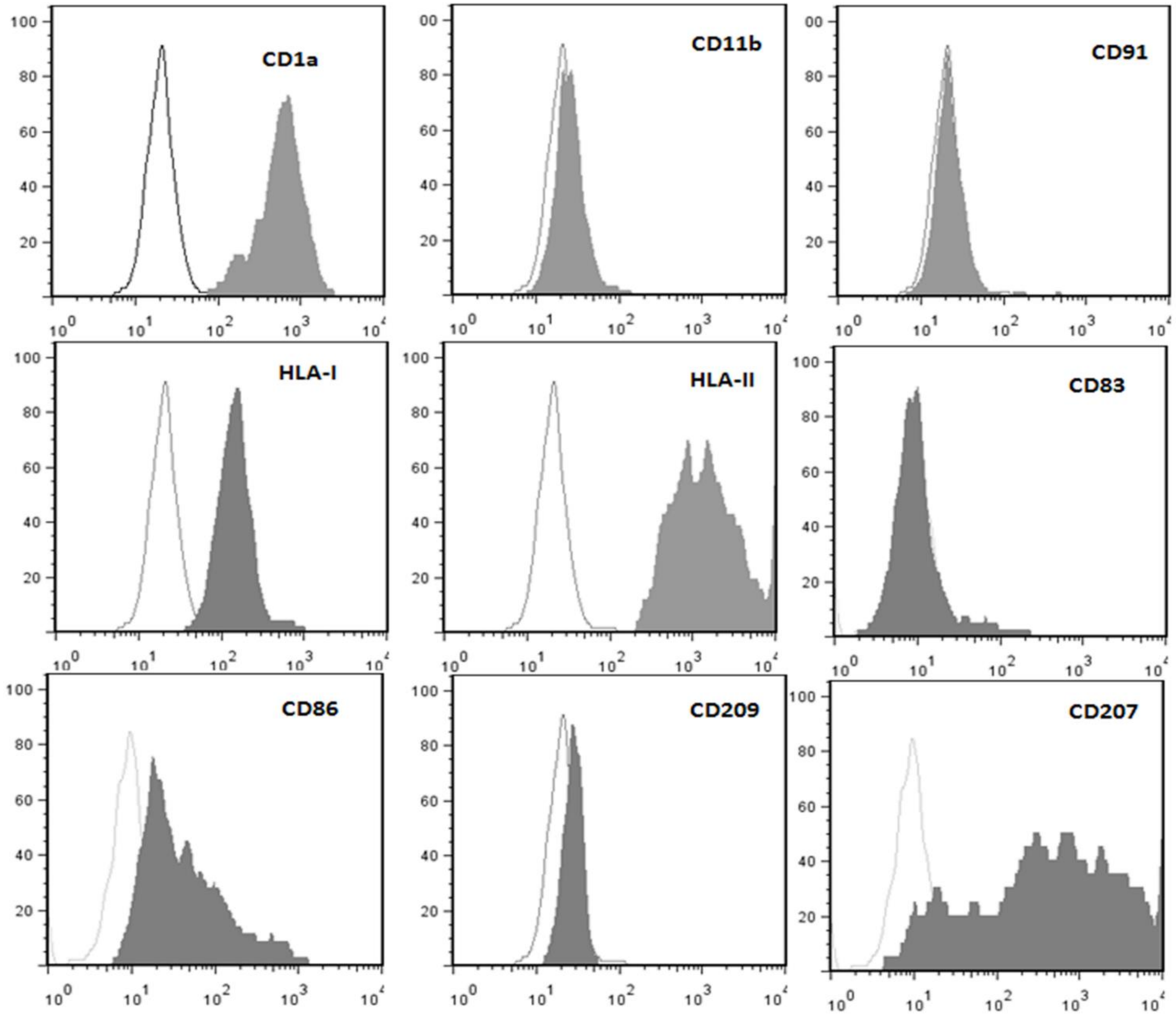


**Figure S1. Phenotypic characterization of CD1a+ sorted LC.** CD1a+ cells were sorted with CD1a mAb coated, immunomagnetic beads and stained for surface marker expression as described in Materials and Methods. Empty histogram: isotype control; filled gray histogram: mAb stained cells.

**Figure S2**

LC

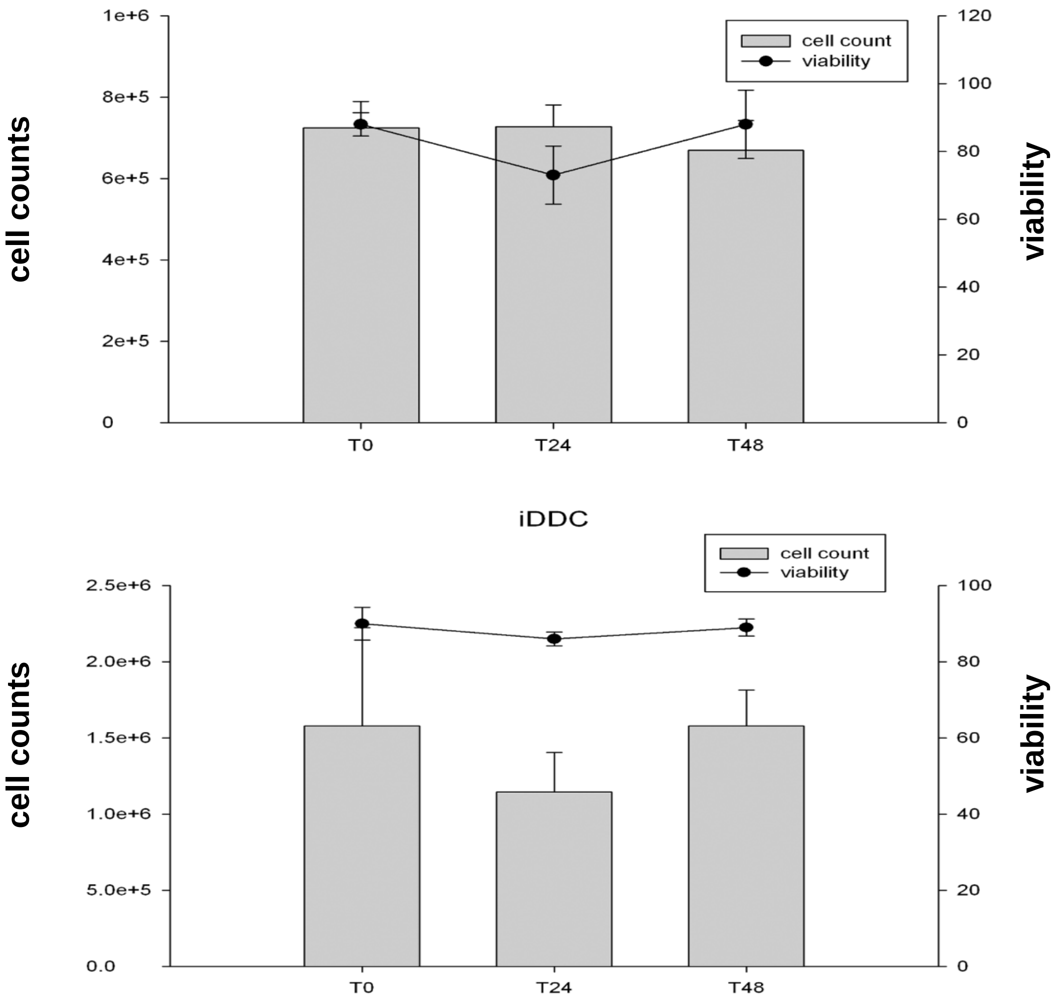


**Fig.S2. Cell counts and viability in infected iDDC and LC**. Cultured cell were collected at the indicated time points, counted and viability was determined by trypan blue exclusion.

Figure S3

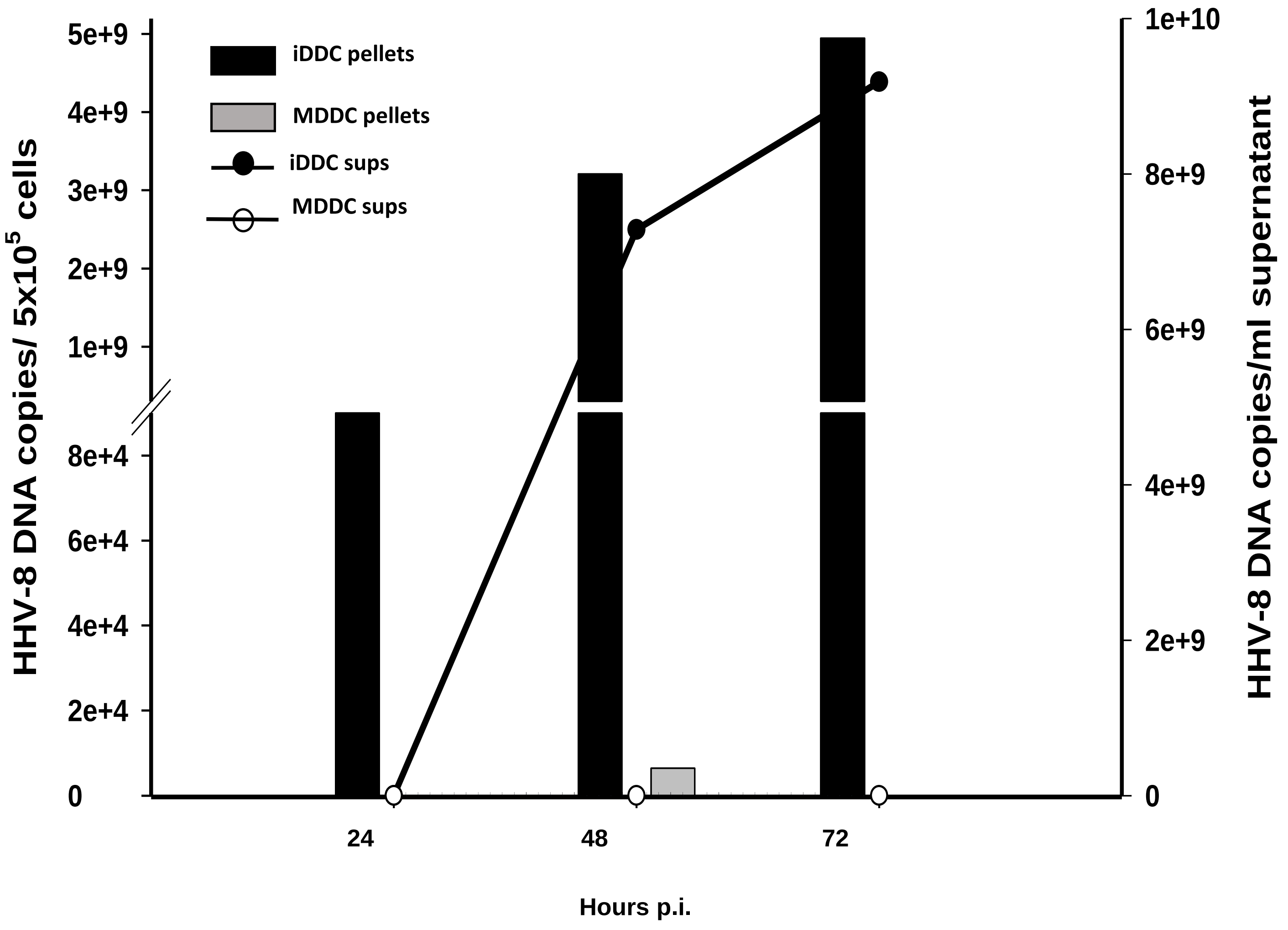


**Fig. S 3**. **HHV-8 does not replicate in neonatal MDDC**. Quantitative DNA results from real time PCR assay of total DNA that was extracted from infected iDDC and MDDC cell pellets (black and grey bars, respectively) and the corresponding DNAse treated cell culture supernatants (black and grey line, respectively).

## Fig. S4

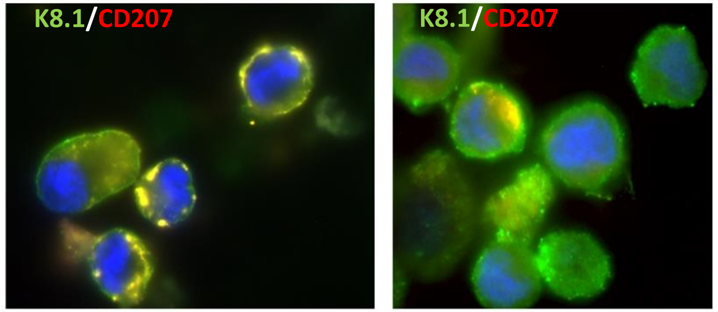


**Fig. S5. HHV-8 infection of LC is partially blocked by anti-CD207 mAb**: A: LC were left untreated (left panel) or treated with anti-CD207 moAb (R&D Systems clone 343828, 20 ug/ml) prior to infection with HHV-8. Cells were stained with anti-CD207 (red) and anti K8.1A/B (green)

Fig. S5

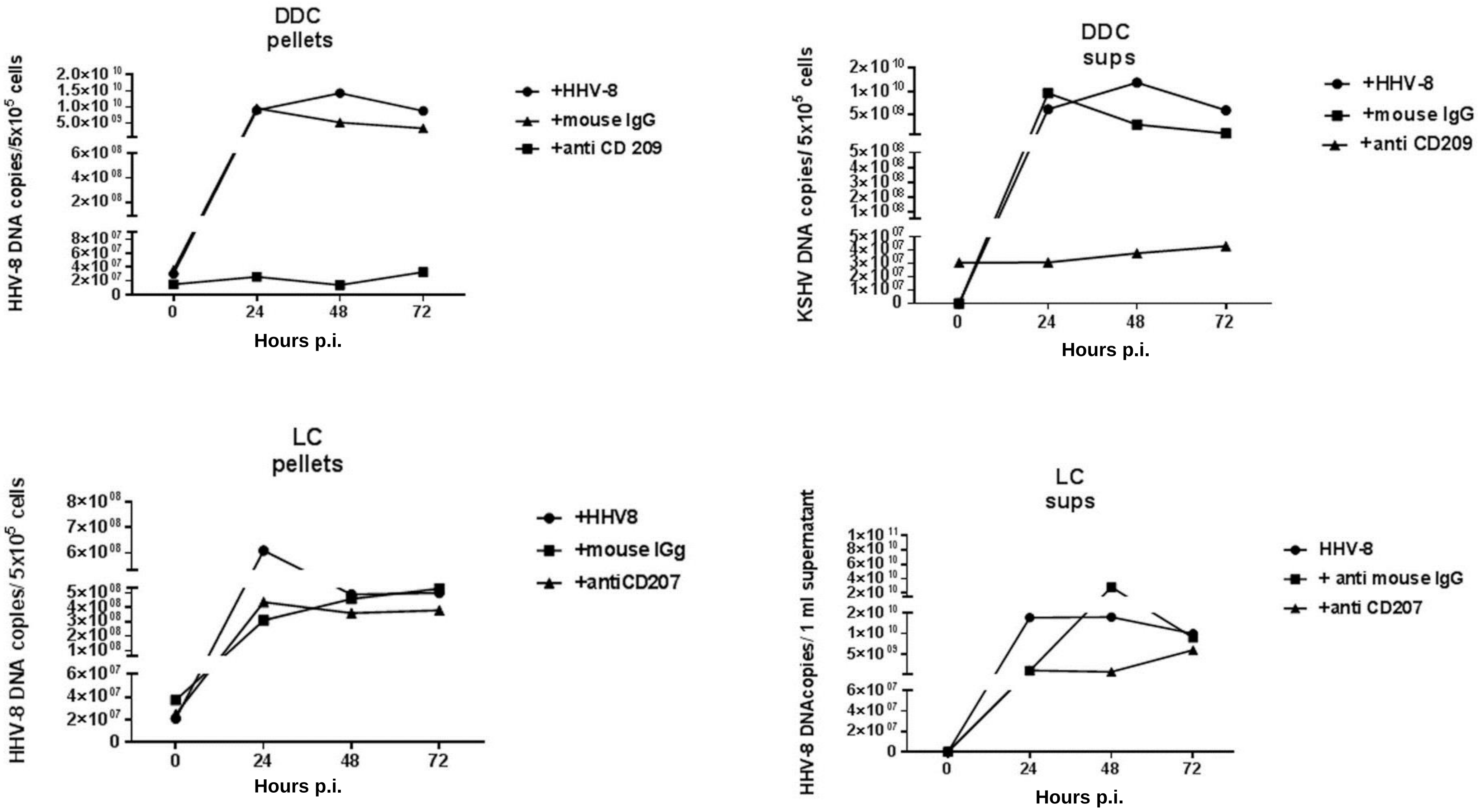


**Supplemental Figure 5**. HHV8 infection of iDDC is blocked by anti CD209 moAb, but not by anti-CD207 in Langerhans cells. iDDC or LC were treated with anti-CD209 or-CD207 prior to infection with HHV-8 as described in Materials and Methods. Cells incubated with mouse IgG were used as control.
